# Comorbidity of diabetes mellitus in idiopathic normal pressure hydrocephalus: a systematic literature review

**DOI:** 10.1186/s12987-019-0125-x

**Published:** 2019-02-12

**Authors:** Miles Hudson, Caden Nowak, Richard J. Garling, Carolyn Harris

**Affiliations:** Department of Neurosurgery, Wayne State University, 51 W Palmer ave, Detroit, MI 48202 USA

**Keywords:** iNPH, NPH, DM, GH, IGF-1

## Abstract

Idiopathic normal pressure hydrocephalus (iNPH) is a subtype of hydrocephalus that occurs more often in the elderly population. It is usually characterized by gait disturbance, dementia and urinary incontinence. Epidemiological studies indicate that 15.7–17.8% of iNPH patients present with type-2 diabetes mellitus (DM). A review of the primary literature shows that these occurrence rates are higher than age- and cohort-matched non-iNPH controls. This suggests that this already vulnerable patient group has an increased risk for presenting with DM compared to their non-iNPH counterparts. Postoperative outcome when treating iNPH patients is inversely related to the number of patient comorbidities and a lower comorbidity status is correlated with better outcomes. This review highlights the need for further research into the relationship between iNPH and DM and speculates on a possible mechanism for an association between the development of ventriculomegaly and the development of DM and iNPH.

## Introduction

Normal pressure hydrocephalus (NPH) is a subtype of hydrocephalus that has an incidence of 181.7 per 100,000 patients between the age of 70–79, with more than 50% of NPH patients being diagnosed after the age of 70 [1]. There are two categories of NPH: idiopathic and secondary. Idiopathic NPH (iNPH) often presents as a spontaneous rise in ventricular size; secondary NPH arises as a complication to another condition such as subarachnoid hemorrhage, tumor, or traumatic brain injury (TBI) [2]. The physiologic manifestation of the condition is best described as a slow accumulation of cerebrospinal fluid (CSF) in the ventricles leading to a gradual increase in stiffening and increase in ventricular size with a normal intracranial pressure. This results in a normal CSF pressure measured downstream in the system via lumbar tap [3]. In both iNPH and secondary NPH, ventriculomegaly leads to a triad of common symptoms. These include gait disturbances, dementia, and urinary incontinence [4]. The most common treatment for the condition is shunt-based surgery, in which a shunt system is inserted into the cerebral ventricles or lumbar subarachnoid space, draining excess CSF to different regions of the body depending on shunt type.

Patient outcome post-shunt therapy has been shown to be inversely related to the number of comorbidities presented and a favorable outcome post-shunt therapy decreases as the number of comorbidities increases [5]. Comorbidity-related iNPH studies in the past, more often than not, have focused on hypertension, but epidemiological studies have shown that nearly 18% of iNPH patients present with diabetes mellitus (DM) [6]. Identifying and managing the risk factors associated with all comorbidities is crucial for optimal patient outcome. iNPH and some other forms of hydrocephalus are known to be associated with endocrine and pituitary dysfunction. Growth hormone (GH) is secreted by the anterior pituitary gland and induces the secretion the downstream effector molecule insulin-like growth factor 1 (IGF-1) from the liver [7]. Altered levels of GH and IGF-1 have been associated with insulin resistance and the development of diabetes mellitus. IGF-1 is involved in glucose homeostasis affecting insulin levels, fat/muscle glucose uptake and hepatic glucose output [8].

This literature review seeks to examine the association between iNPH and DM and has two primary objectives. The first is to investigate the relationship between DM and iNPH to determine if iNPH patients are more likely to present with DM compared to their non-iNPH counterparts. The second is to examine the literature to determine if a potential independent mechanism involving ventriculomegaly, iNPH, and pituitary dysfunction is plausible and could explain the relationship. Investigating mechanisms for the association between DM and iNPH may assist in the monitoring and treatment of patients who present with iNPH and DM.

## Methods

For all searches conducted, non-English, unavailable, and non-published studies were excluded. A PubMed search was performed using the phrase *Epidemiology of idiopathic Normal Pressure hydrocephalus* to look at the co-morbidity rates associated with iNPH. The search returned 197 reviewable articles. Studies including comorbidities associated with iNPH patient populations were reviewed. Two studies met this refined criteria. A second PubMed search was performed using the term *diabetes and normal pressure hydrocephalus.* A total of 32 articles were found. Studies not comparing DM occurrence in an iNPH group to a non-iNPH group were excluded, resulting in seven studies meeting the criteria.

Another PubMed search was performed using *pituitary function and hydrocephalu*s identifying 169 studies of interest. Studies not focusing on hydrocephalus as an independent variable were excluded, resulting in eight studies meeting inclusion criteria. A second search was performed using the terms *hypopituitarism and hydrocephalus*, resulting in 70 studies. The same exclusion criteria were used resulting in two additional studies being identified for a total of 10 studies included in the review. In addition, select literature sources were examined to determine if there was a relationship between decreased growth hormone/IGF-1 levels and the development of diabetes mellitus. To reduce bias, a source number of n = 7 relating to altered GH/IGF-1 levels and DM was used.

## Results

Of the two studies from our search for *Epidemiology of idiopathic Normal Pressure hydrocephalus*, the first was a Japanese nationwide study looking at the patient data of 12,900 iNPH patients. The study found that 40% of the iNPH patients had hypertension, 17.8% had diabetes and 14.8% of patients had Alzheimer’s disease [6]. The second study was based in Norway comparing the iNPH population to the HUNT3 survey. The HUNT3 survey was a nationwide collection of medical data from patients in Nord-Trøndelag Norway over the age of 13. The study found the two highest comorbidities to be hypertension with a rate of 41.8% and DM with a rate of 15.7%. Both conditions appeared significantly more frequently in the iNPH group than the control group (P < 0.001) [9].

**Fig. 1:**
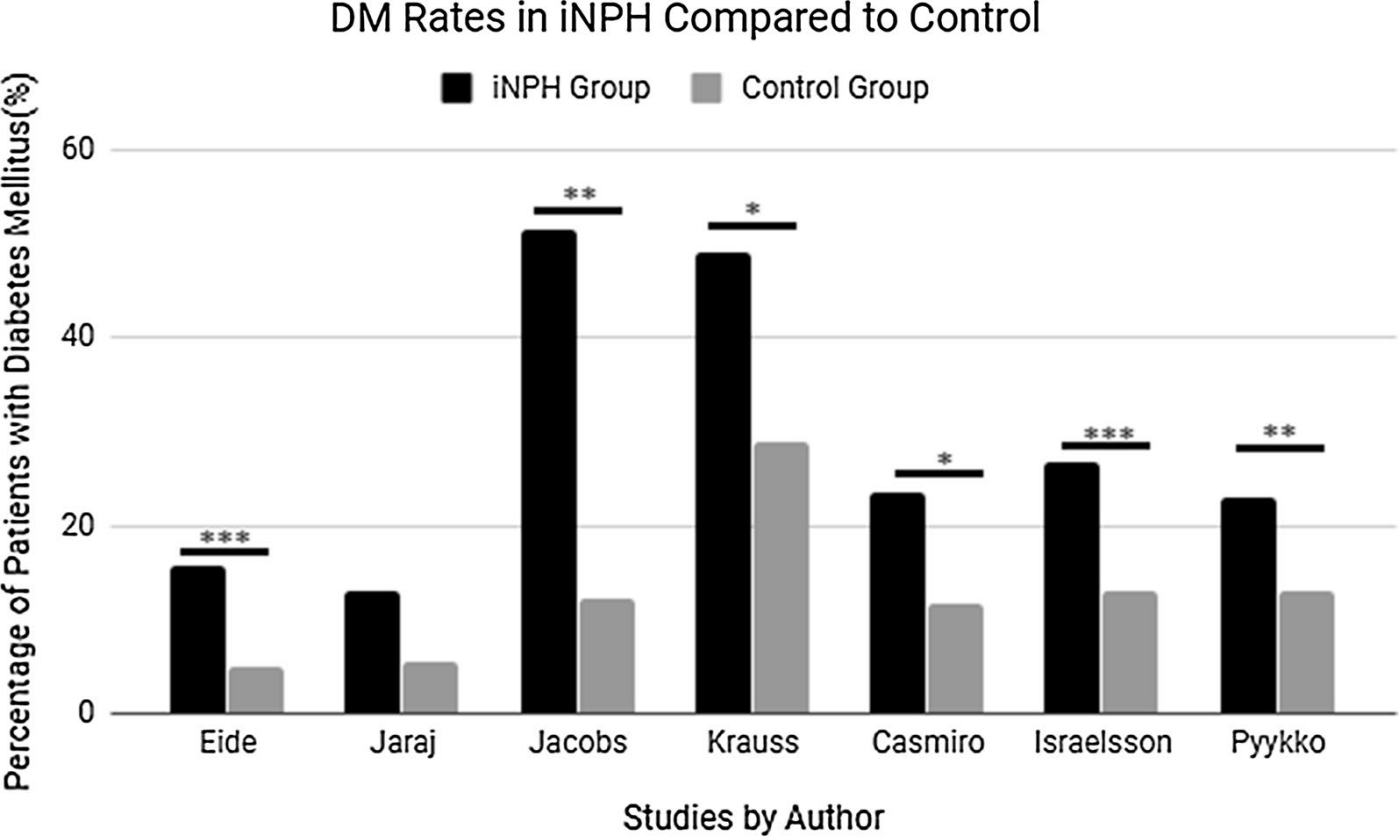
DM rates in iNPH group compared to control. The percentage of patients in each case controlled study presenting with DM in either the iNPH group or the study’s control group (varying by study). Number of patients in each group: *N* iNPH, *C* control. Eide: N-440, C-43,387, Jaraj: N-26, C-130, Jacobs: N-33, Kraus: N-63, C-70, Casmiro: N-4, C-2 Israelsson: N: 38, C-44, Pyykko: N-283, C-253. Studies indicated with a “*” had statistically significant results P < .05, “**” = P < .01, “***” = P < .001 (error bars were unavailable for the above studies)

Seven studies were found comparing DM occurrence rates in an iNPH group to a non-iNPH and age-matched control group (Fig. 1). The first study (Eide et al.) comparing 440 iNPH patients with 43,387 control patients matched for age and sex, found a statistically significant increase in DM occurrence in the iNPH group of 15.7% compared to 5% in the control group (P < 0.001) [9]. The second study (Jaraj et al.) compared 26 suspected iNPH patients to 130 age-, sex-, and cohort-matched non-iNPH control patients, with fivefold more controls per iNPH patient. The authors found a non-significant trend indicating an increase in DM, with a 13% occurrence rate in the iNPH group compared to 7.6% in their control group [10]. The third study (Jacobs et al.) compared a group of 33 patients presenting with iNPH to non-iNPH controls, and found a statistically significant increase in DM occurrence in the iNPH patient group. DM rates were 51.5% compared to 12.1% respectively (P < 0.01) [11]. The fourth study (Krauss et al.) compared 63 iNPH patients to 70 age-matched non-iNPH controls. There was a statistically significant increase in DM occurrence in the iNPH group of 49% compared to 29% in the control group (P < 0.015) [12]. The 5th (Casmiro et al.) and 6th (Israelsson et al.) studies also compared DM occurrence rates in iNPH patients to controls and found the occurrence rates to be 23.5% and 26.8% compared to 11.8% and 13.1% respectively (P < .018, P < .001) [13, 14]. The final study (Pyyko et al.) compared 283 iNPH patients to 253 non iNPH patients and found the DM rate in the iNPH group to be 23% compared to 13% respectively (P < .002) [15].

Ten studies were found examining pituitary function as it related to hydrocephalus with three focusing on iNPH specifically (Table 1). When analyzing each study our focus was directed towards patients growth hormone levels. When this information was not available and when present the patient IGF-1 levels found by the authors were recorded. When IGF-1 and GH levels were not available the effects on pituitary function were recorded. Of the identified studies three were cross-sectional studies and found a statistically significant decrease in pituitary function or growth hormone/IGF-1 levels (*P* < 0.05) (Table 1).

**Table 1:** Effects of hydrocephalus on pituitary function

Year	Author	Type of hydrocephalus	Pituitary function findings	Type of study
2012	Moin et al. [16]	*iNPH*	*Decreased IGF*-*1*	*Experimental study*
2011	Pinto et al. [17]	*Chronic hydrocephalus*	*Decreased growth hormone*	*Single case study*
1997	Löppönen et al. [18]	Shunted hydrocephalus	Decreased growth hormone***	Controlled cross sectional study
1998	Löppönen et al. [19]	Shunted hydrocephalus	Decrease in growth hormone and IGF-1 post shunting	Case study
1977	Hier et al. [20]	*Chronic hydrocephalus*	*Decreased growth hormone*	Multiple *case study*
2016	Vieira et al. [21]	Post hemorrhagic acute phase hydrocephalus	Patients with hydrocephalus had increased pituitary dysfunction when compared to non-hydrocephalus patients***	Cohort study
1978	Barber et al. [22]	*iNPH*	*Decreased growth hormone*	*Single case report*
1979	Barber et al. [23]	iNPH	Hypopituitarism	Multiple case report
1997	Hochhaus et al. [24]	*Hydrocephalus*	*Decreased IGF*-1***	*Cross sectional study*
2015	Khajeh et al. [25]	Post hemorrhagic hydrocephalus	Pituitary dysfunction and growth hormone deficiency	*Case study*

Studies examining the effects of multiple types of hydrocephalus on pituitary function specifically related to growth hormone/IGF-1. Pituitary function findings indicated with (***) indicate a statistically significant result compared to age and cohort matched controls

Seven select sources are listed below citing low GH/IGF-1 levels and their association with diabetes mellitus (Table 2). All but two of the seven studies show statistically significant data (*P *< 0.05).

**Table 2:** Growth hormone/IGF and diabetes

Year	Author	Findings	Type of study
2010	Teppala et al. [26]	Low serum IGF-1 levels were positively associated with diabetes***	National survey
1996	Hew et al. [8]	Decreased insulin sensitivity, decreased glucose storage rate, decreased glycogen synthase***	Controlled cross sectional study
2010	Schneider et al. [27]	Low or high serum levels of IGF-1 are associated with diabetes	Multiple prospective cohort studies
2005	Murray et al. [28]	Growth hormone resistant and insufficient adults are insulin resistant when stimulated with insulin***	Controlled cross sectional study
1984	Amiel et al. [29]	Low lGF-1 levels are positively associated with diabetes***	Controlled cross sectional study
2004	Jørgensen et al. [30]	Growth hormone deficient adults tend to be insulin resistant	Multiple case study
1995	Johansson et al. [31]	Adults with growth hormone deficiency were found to have normal fasting plasma insulin levels and insulin resistance***	Controlled cross sectional study

Select sources related to growth hormone deficiency in adults and it’s associated to diabetes mellitus. Studies indicated with (***) involve an age and cohort matched non-iNPH control group and a statistically significant result

## Discussion

Epidemiological studies have found DM occurrence rates in the iNPH patient population to range from 15.7 to 17.8% [6, 9]. Determining if the occurrence of DM is ‘normal’ or if iNPH patients are at increased risk was the next logical step. Review of the available literature after exclusion, indicated seven studies directly comparing DM occurrence rates between iNPH and non-iNPH patient populations (Fig. 1) [9–15]. Five of these were found to have statistically significant increases in DM in the iNPH patient population over control with the 6th study having a non-significant increase. This supports the hypothesis that the iNPH patient population is at a greater risk for presenting with DM than their non-iNPH counterparts. The majority of the studies examined compared the iNPH group to a non-iNPH group with age- and cohort-matched controls. This indicates that the iNPH patients are not presenting with a higher DM occurrence simply due to the fact that the elderly iNPH patient demographic is already at elevated risk for DM. These results are vital because they show statistical significance and that the increase in occurrence is not trivial, with the smallest statistically significant result being a 10% difference in the DM occurrence rate. These results set the stage for further research and larger scale studies by demonstrating the possibility that the DM may be a risk factor or early indicator of possible ventricular dysfunction and development of iNPH in the elderly population. Future studies are necessary and should focus on following a cohort of iNPH patients for hormone levels, development of DM, and ventricular and brain imaging to look at the relevant factors discussed in this study.

iNPH patients with high comorbidity status are at an increased risk for unfavorable postoperative outcomes, but DM itself also introduces its own specific complications such as diabetic neuropathy, retinopathy, nephropathy, and increased risk of vascular disease such as atherosclerosis. These complications can be quite severe in the already elderly patient population in which ventriculomegaly and iNPH are more likely. We seek to propose an independent mechanism relating DM to iNPH in the hopes of developing a more specialized treatment plan and prevention method for the condition.

**Fig. 2:**
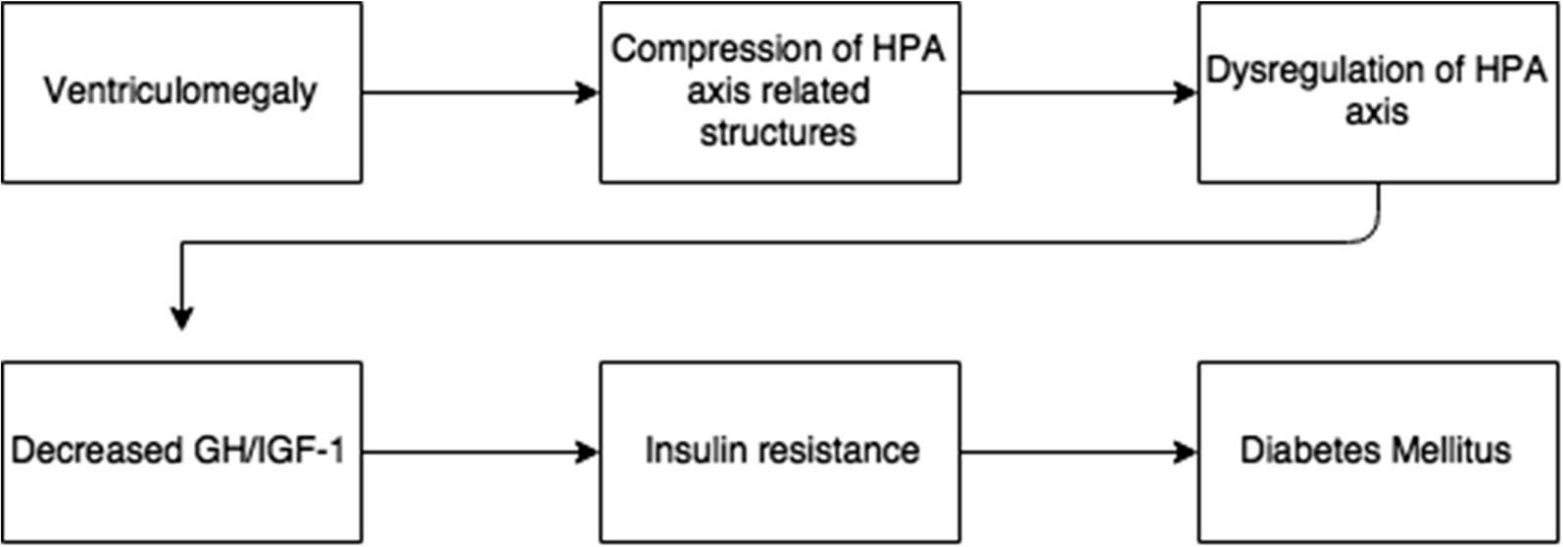
Hypothetical association between ventriculomegaly and diabetes mellitus. Proposed mechanism for ventriculomegaly, and the subsequent development of diabetes mellitus. *HPA* hypothalamic pituitary axis, *GH* growth hormone, *IGF-1* insulin-like growth factor

iNPH as well as other forms of hydrocephalus have been shown to affect the hypothalamic pituitary axis (HPA axis) [16, 21]. Though the exact mechanism of the effect is still under debate, our review of the literature supports the claim that ventriculomegaly and iNPH specifically can be associated with HPA axis dysregulation (Table 1). This dysregulation may be a result of ventriculomegaly causing compression and dysfunction of key structures in the HPA axis such as the hypothalamus, pituitary gland, or surrounding vasculature. When it comes to dysregulation of the HPA axis one of the most vulnerable and commonly affected axis is the GH axis [32]. Dysregulation of this axis can lead to decreased levels of circulating GH resulting in a decrease in IGF-1 secretion from the liver. IGF-1 increases glucose uptake in fat/muscle, inhibits hepatic glucose output, and suppress insulin secretion from the pancreas, with decreased levels having the opposite effect [7]. Though the exact mechanism has not been defined, many studies have found a correlation between IGF-1 deficiency and the development of insulin resistance [8, 31]. Patients with a deletion in the IGF-1 gene have been shown to develop insulin resistance that improved with IGF-1 therapy [33]. These findings in combination with those data reviewed here (Table 2) highlight the proposed connection between a decrease in GH/IGF-1 and the development of DM (Fig. 2). It is plausible that ventriculomegaly and compression of nearby structures causes the triad of iNPH symptoms as well as the symptoms of DM. Future studies conducted could evaluate the timeline further to describe the presentation of symptoms of the of the two conditions. Based on these data, GH or IGF-1 replacement should be further examined as a possible treatment avenue for patients presenting with DM due to compression of underlying structures. Further study is needed to determine the efficacy of this proposed treatment method.

## Conclusion

Idiopathic normal pressure hydrocephalus is a complicated condition with a number of associated comorbidities, one of the most prevalent being diabetes mellitus. This review highlights the increased occurrence of DM in the iNPH population compared to age- and cohort-matched non-iNPH controls. Reducing the comorbidity status of iNPH patients is crucial when it comes to improving patient outcomes post-shunt therapy. This likely involves evaluation of the patient population as well as the development of future specialized treatments. To develop these treatments the mechanisms underlying ventriculomegaly and the development of DM need to be better understood. Based on literature review only we can speculate that ventriculomegaly-induced pituitary dysfunction causing the subsequent development of DM is possible, and may explain the increased rates of DM in the iNPH population.
